# Initial response of phenology and yield components of wheat (*Triticum durum* L., CIRNO C2008) under experimental warming field conditions in the Yaqui Valley

**DOI:** 10.7717/peerj.5064

**Published:** 2018-06-21

**Authors:** Jaime Garatuza-Payan, Leandris Argentel-Martinez, Enrico A. Yepez, Tulio Arredondo

**Affiliations:** 1 Instituto Tecnológico de Sonora, Cd. Obregon, Sonora, Mexico; 2 Instituto Potosino de investigación Científica y Tecnológica, San Luis Potosi, San Luis Potosi, Mexico

**Keywords:** Photosynthesis, Stress tolerance, Temperature increase, Stress intensity, CIRNO2008

## Abstract

This work evaluates the experimental warming effects on phenology and grain yield components of wheat in the Yaqui Valley, Sonora, México, using CIRNO C2008 variety from *Triticum durum* L., as a model during the cropping cycle of 2016–2017 (December to April). Infrared radiators were deployed to induce experimental warming by 2 °C above ambient crop canopy temperature, in a temperature free-air controlled enhancement system. Temperature was controlled by infrared temperature sensors placed in eight plots which covered a circle of *r* = 1.5 m starting five days after germination until harvest. The warming treatment caused a reduction of phenophases occurrence starting at the stem extension phenophase. Such phenological responses generated a significant biological cycle reduction of 14 days. Despite this delay, CIRNO C2008 completed its biological cycle adequately. However, plant height under the warming treatment was reduced significantly and differences were particularly observed at the final phenophases of the vegetative cycle. Plant height correlated negatively with spikes length, spikes mass, and number of filled grains. Warming also reduced grain yield in 33%. The warming treatment caused a stress intensity (SI = 1-yield warming/yield control) of 39.4% and 33.2% in biomass and grain yield, respectively. The differences in stress intensities between biomass and grain yield were based on plant height reduction. Grain mass was not affected, demonstrating the crop capability for remobilization and adequate distribution of elaborated substances for the spikes under warming conditions.

## Introduction

The biological cycle of plants is frequently affected by several climatic and edaphic factors (Chuine & Beaubien, 2001; Ahmed et al., 2017). Reduction of phenophase time generally produces irreversible effects and involves biochemical and physiological modifications directly correlated with yield decrease (Singh et al., 2017). It is common to find considerable variability in phenophase time among species in response to warming (Wang et al., 2017), but the magnitude of the effect is not always correlated with yield. Photosynthetic activity, the most important process in plants, for example, does not always contribute to yield increase by increasing its rate when stress intensity is higher than the varietal tolerance (Blum, 2011).

Warming stress is a common abiotic factor generally overlooked by producers (Siebert et al., 2017); however, high temperatures modify a plant’s hormonal and gene expression, reducing the phenophase occurrence time with significant yield affectations. Most phenophases require a certain time for a normal development (Valluru et al., 2017). Wheat, for example, requires the accumulation of certain amount of heat units (cold hours) to complete its normal phenology (Zadoks, Chang & Konzak, 1974; Argentel Martínez et al., 2017). The completion of this accumulated heat (physiological time) involves the appropriate combination of temperature degrees, that determines Tillering capacity, and its relation with tiller growth (Solanki et al., 2017), which sometimes depletes yield when its occurrence time is reduced (Asseng et al., 2017).

It is known, in high wheat producing regions, that temperature is a potential threat for production (IPCC, 2014), as confirmed by parameterized models (Ahmed et al., 2017); however, the effect of warming on plant physiology is not yet clearly explained in highly wheat producing regions. Every year, thousands of hectares of crops are abandoned because plants do not overcome warming stress and cannot, therefore, express, completely, their genetic productive potential (Trnka et al., 2017; Gouache et al., 2017).

Temperature increments of around 2 °C have been predicted for several latitudes over the next 50 years. These areas include the semiarid region of Mexico where wheat is produced (Garatuza-Payán et al., 1998). Monitoring the phenological and agronomical variability response of wheat under field conditions, with the use of precise technologies to verify the temperature increase effect, will allow to explain possible mechanisms involved in phenological warming response and its relationship with biomass and grain yield (Dettori, Cesaraccio & Duce, 2017). The present research, which constitutes the first study in Mexico and Latin America, under field conditions, evaluated the temperature increase effect, by about 2 °C on crop canopy, in the phenology and grain yield components of wheat (*Triticum durum* L.) in the Yaqui Valley, Sonora, Mexico. This region produces almost 40% of the national wheat production and will potentially be affected by climate change (Cavazos & Arriaga-Ramírez, 2012).

## Material and Methods

The experiment was carried out during the 2016–2017 cropping cycle (December to April) in 16 plots (eight Warming and eight Control) under field conditions, at the Experimental Technology Transfer Center (CETT-910) of the Instituto Tecnológico de Sonora (ITSON), located in the Yaqui Valley at: 27°22′0.4″ N and 109°54′50.6″ W (UTM: 607393.24 m E; 3027508.34 m N).

### Treatments and temperature control

In order to study the wheat response to increased temperature, two treatments of eight replicated plots (*n* = 8) were established: T1: increased canopy temperature aimed at 2 °C with respect to ambient canopy temperature of adjacent plots (Warming treatment); and T2: at ambient canopy temperature (Control treatment).

**Figure 1:**
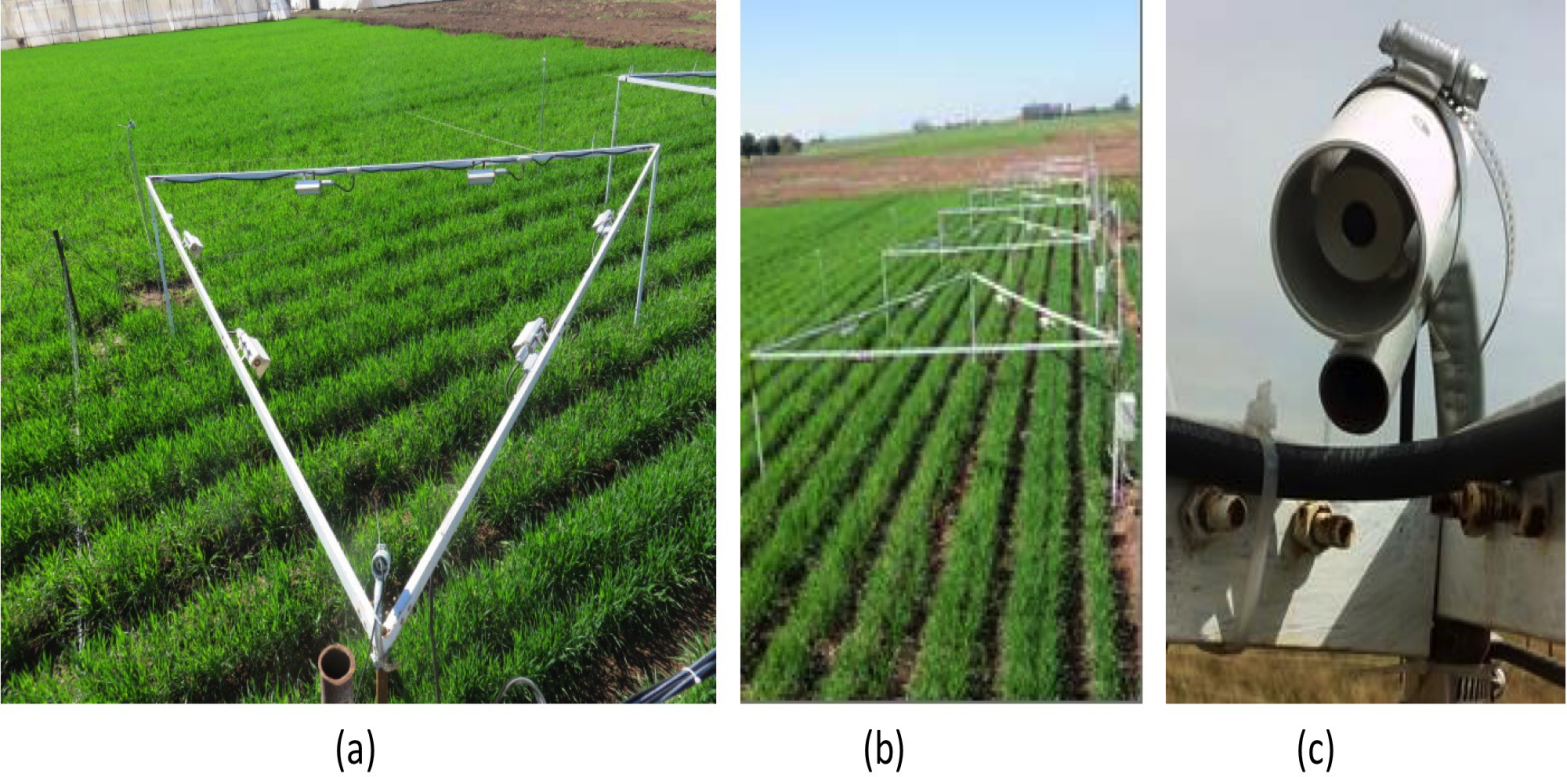
(A) six radiators by structure, (B) eight triangular structures, and (C) infrared sensor. Source: the authors.

The experiment consisted in raising crop canopy temperature by 2 °C, using a temperature free-air controlled enhancement system. A total of six thermal radiators per plot (FTE-1000 model, 1,000 W, 240 V, 245 mm long × 60 mm wide, built by Mor Electric Company Heating Association Inc. Comstock Park, MI, USA), (Kimball, 2015) were installed on eight equilateral triangular structures of 5.2 m of side. A total of two radiators were mounted on each side of the triangular structures (Fig. 1) forming a regular hexagon which effectively raised the temperature by 2 °C on a 3 m diameter circular plot. To control temperature, infrared temperature sensors (IRTS Apogee Instruments Inc., Logan, UT, USA) were installed on each plot at an inclination of 45° from the horizontal surface, to cover an ellipse of 3 m in the major axis at the center of the plot. The IRTS signal was received in a datalogger (CR1000 Campbell Sci, Inc., Logan, UT, USA), which sent a voltage signal to an interface (MAI-05V; Avatar Instruments, Lewes, DE, USA) that translated the voltage signal to milliamps to control a regulator (Dimmer A1P-24-30-S05; Avatar Instruments, Lewes, DE, USA). This regulator controls the current sent to the heaters, so that the amount of emitted heat by them increases or decreases as a function of the temperature difference between the Warming plot and the Control plot, through the proportional, integrative, and derivative routine described in Kimball (2015). The electronic system was programmed to keep a constant temperature of 2 °C in the Warming treatments, above the reference plot temperature. However during the heading phenophase the system struggled to reach 2 °C between 11:00 and 14:00 h local time, particularly during windy days, inducing only 1.3–1.4 °C of warming over these periods, thus simulating the temperature increase predicted for this region for the year 2050 according to the IPCC (2014).

### Variety used as a model, seeding, and cultural practices

CIRNO C2008 wheat variety is classified as crystalline or hard wheat (*T. durum* L.). It was originated from selection in segregating populations of SOOTY-9 / RASCON-37 // CAMAYO crossbreed, carried out at the International Center for of Maize and Wheat Improvement. This variety was released for cropping since 2008, being widely used in Mexico and, particularly, in Sonora. It has a spring growth habit, ideal to be cropped during the autumn–winter cycle. Under irrigation, the spike appears from 74 to 89 days and physiological maturity is reached at 122 days. CIRNO C-2008 has an average height of 78 cm with erect stems. Before maturing, the sheath of the flag leaf and the peduncle of the spike have strong and visible levels of wax. Grain yield when the variety was released in Sonora reached 5.6 and 6.3 t ha^−1^ with two and three irrigations, respectively (Figueroa-López et al., 2010).

Seeding was done with a sowing machine (SUB-24) on December 8th, 2016 on a vertisol soil (Wilding, Smeck & Hall, 1983), with three rows on the furrows and a seeding density of 170 kg ha^−1^. Background fertilization was done to a standard of 250 kg ha^−1^ of urea + 100 kg ha^−1^ of monoammonium phosphate, 11-52-00. During the first irrigation (January 25, 2017 Growth phenophase), nitrogen fertilizer was applied at a dose of 50 kg ha^−1^ of urea. In the second irrigation (February 20, 2017, Tillering phenophase) nitrogen fertilizer was applied at a dose of 50 kg ha^−1^ of urea. In the third irrigation (March 15, 2017, Heading phenophase) there was no fertilizer application. All irrigations were applied with an average water depth of 14 cm each (1,400 m^3^ ha^−1^) and at an interval of about 25 days. All irrigations were applied when gravimetric water volume of the soil was at about 70% measured with a theta probe (Delta-T Devices, Cambridge, UK) following the recommendations of the local agricultural research service (Figueroa-López et al., 2010).

### Pest, diseases, and weed control

Pests, diseases, and weeds were controlled by appropriate chemical applications. During the growing period the presence of foliage aphid (*Schizaphis graminum*) was found and *Muralla Max* (a.i. Imidacloprid + Betaciflutrin) was applied at a rate of 0.20 L ha^−1^ on the periphery of the plot to 2–3 m from the border surface. The pesticide was applied, at Tillering phenophase. Likewise, a slight presence of broadleaf weeds was observed and they were controlled by manual weeding before applying irrigations.

### Phenological response

Phenological response was studied using the Zadoks comparative decimal scale (Zadoks, Chang & Konzak, 1974), considering a phenophase when more than 50% of the population showed the related characteristics. The phenophases evaluated were: Z0: Germination; Z1: Growth, Z2: Tillering, Z3: Stem elongation; Z4: Booting; Z5: Heading, Z6: Flowering; Z7: Milk development; Z8: Dough development, and Z9: Maturity.

Phenophase occurrence time was evaluated between Warming and Control treatments by comparing: days at growth (2–3 and 4–7 Leaves); days at tillering; days to the appearance of the first node; days to the appearance of the second node; days to booting, days to the heading; days to flowering; days to grains filling (Milk and Dough grain), and days to physiological maturity.

### Photosynthetic activity

Instantaneous maximum photosynthetic rate (A, μmol CO_2_ m^−2^s^−1^), was measured during all evaluated phenophases in leaves well exposed to direct radiation between 11:00 and 13:00 h on sunny days. For this measurement, a portable photosynthesis system (LI-6400XT; LI-COR, Inc., Lincoln, NE, USA) was used. The three leaves most exposed to direct radiation (three repetitions per plant), were inserted, by its central part, into the natural light gas exchange chamber of 3.0 × 2.0 cm, which is coupled to a portable infrared gas exchange system. All measurements were done with a light intensity over 1,500 μmol m^−2^ s^−1^, and with a constant CO_2_ concentration of 400 μmol mol^−1^ in the reference cell, at a constant flux rate of 500 μmol s^−1^.

### Yield components

The agronomic behavior was evaluated according to the following variables:

Plants height (cm) was measured from the base of the stem to the apical end of the flag leaf on each phenophase, but only the last one, just before harvesting, was used as yield component. A 3.0-m millimeter tape was used for the measurement. In each treatment 40 plants were sampled. This variable was evaluated from germination to maturity and a developing dynamic was shaped as a function of time.

Spikes length (cm) was measured from the base to the terminal grain with a millimeter ruler of 0.50 m just before harvesting. A total of 40 randomized spikes were taken by treatment. Spike mass (g) was measured on 30 spikes by treatment, with a balance.

The number of full and vain grains by spike (#) was counted after each panicle was carefully minced. A total of 25 panicles were used by treatment.

Grain mass (g) on individual grains was measured with a balance taking a total of 90 randomized grains in each repetition for both treatments. In the same way, thousand grains mass (g) was determined in 10 groups of 1,000 grains by treatment. Total biomass and grain yield (t ha^−1^) were determined in both treatments by harvesting three square meters from each plot for a total of 24 repetitions by treatment.

Stress intensity was determined according to the methodology of Fernández (1993), using the following formula:

$${\rm{SI}} = 1 - \left( {{{{\rm{YW}}} \over {{\rm{YC}}}}} \right)$$

Where YW and YC represent yield average in Warming and Control treatments, respectively.

### Statistical analysis

Theoretical assumptions of normality and homogeneity were verified in the collected data (Kolmogorov, 1933) and the mean and its standard deviation were determined in both treatments. For most evaluated variables, due to the conformation of two single treatments (Warming and Control), statistical processing was based on a hypothesis test between two means for independent samples by variable, and differences were established by means of a theoretical Student’s-*t* probability distribution for quantitative continuous variables for significance levels of *p* < 0.005 and *p* < 0.001 (Gosset, 1917).

For photosynthesis, phenology (time of occurrence), and plant height variables, analysis of variance of simple classification based on a linear model of fix effects (Fisher, 1937) were carried out and differences among phenophases were determined by Tukey post hoc for *p* < 0.001 (Tukey, 1960). Vain grains at basal and distal part comparison was done by means of a double classification analysis of variance, based on a linear model of fix effects. When significant differences in the combination of treatment-spike part source of variation were detected, they were compared by Tukey post hoc for *p* < 0.001. Determination coefficient not adjusted, for treatment and spike part, were determined. For statistical processing, the STATISTICA professional statistical package, version 8.4 for Windows, was used (StatSoft, Tulsa, OK, USA).

## Results

**Figure 2:**
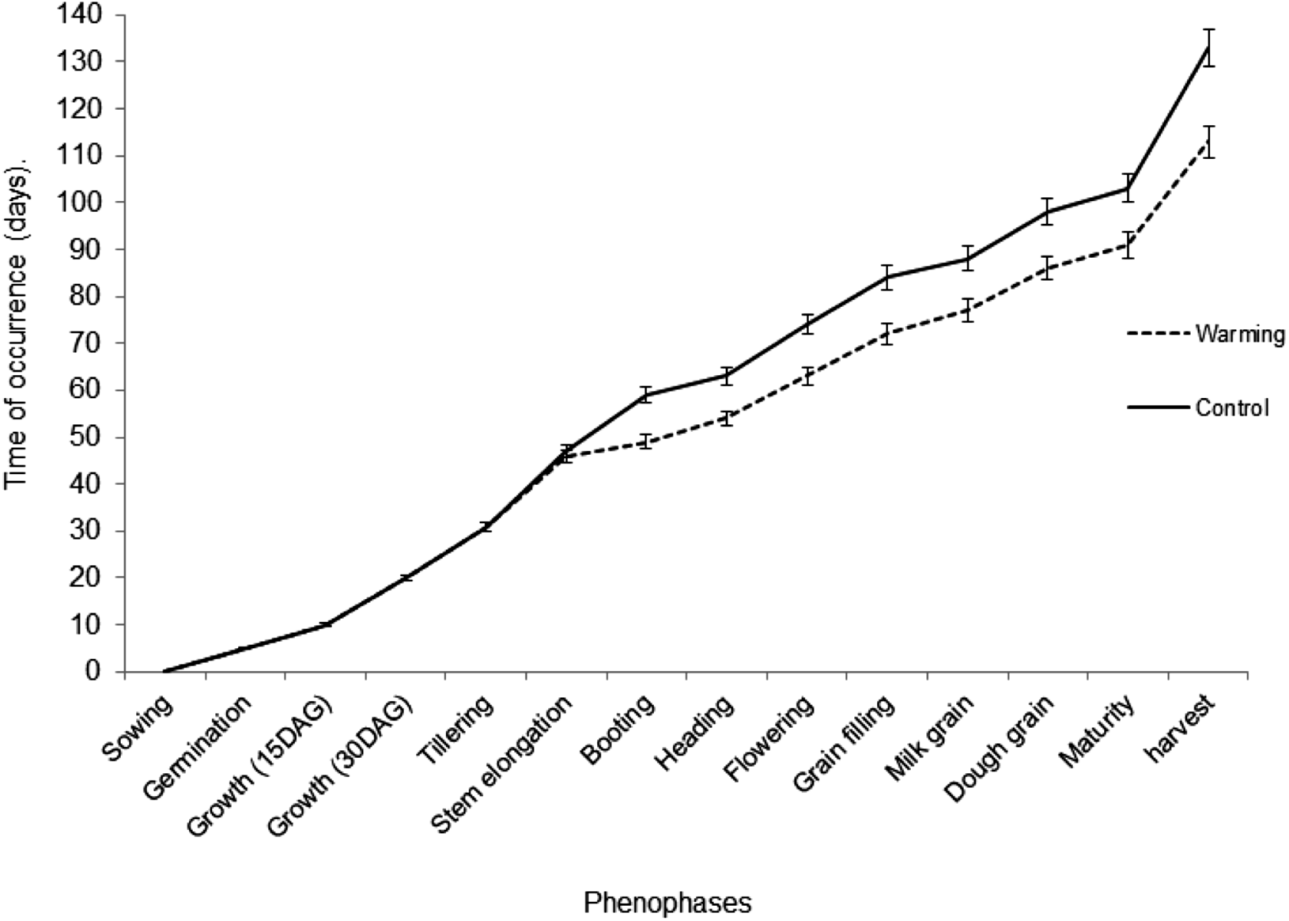
Phenophases occurrence time in both Warming and Control Treatments. Bars for each phenophase represent the standard deviation from the mean.

Temperature increase of the crop canopy did not affect the phenophases occurrence, neither the occurrence time of the initial phenophases of CIRNO C2008 variety, until the second node appearance during Stem elongation. Starting from Stem elongation, over the rest of the cropping cycle, there were highly significant differences in phenology between Warming and Control treatments. The time of occurrence in the Warming treatment was significative lower than the Control. These reductions of phenophase occurrence time were maintained until grain ripening. The greatest differences in the phenophase occurrence time were found during anthesis (10 days) and grain filling (12 days). Such variations generated a difference of 14 days for harvesting. Nevertheless, the studied variety, in both treatments, complied with all phenophases occurrence as describe Zadoks’s decimal comparison scale (Zadoks, Chang & Konzak, 1974) (Fig. 2).

### Yield components

The majority of yield components showed significant differences between Warming and Control treatments, except for grain mass (Table 1). The spike length was one of the most affected variables by warming effect, with a negative and significant contribution to yield decrease (*r* = −0.74, *p* = 0.003). The decrease in the number of full grains per panicle was significative, and its contribution to yield was negative and highly significant (*r* = −0.92, *p* = 0.001). The reduction of full grains number per spike had a great effect on spikes mass and on grain yield and this is attributed as the main cause of yield decrease (*r* = −0.96, *p* = 0.001). On the other hand, vain grains increased in the warming treatment but its contribution to yield decrease was not significant (*r* = −0.38, *p* = 0.29).

**Table 1:** Grain yield components comparison in established treatments.

Treatments	Grain yield components				
	SL (cm)	SM (g)	FG/S	VG/S	MTG (g)
Warming	6.41 ± 0.01	4.43 ± 0.18	43.88 ± 0.5	2.87 ± 0.5	53.1 ± 1.4
Control	7.61 ± 0.02**	5.95 ± 0.07**	51 ± 0.3**	1.87 ± 0.3**	53.2 ± 0.7
*p*	0.003	0.0001	0.0002	0.001	0.132

**Notes:**

SL, spike length, (cm); SM, spike mass; FG/S, full grains per spike; VG/S, vain grains by spike; MTG, mass of 1,000 grains (g).

** Significance at *p* < 0.001.

**Figure 3:**
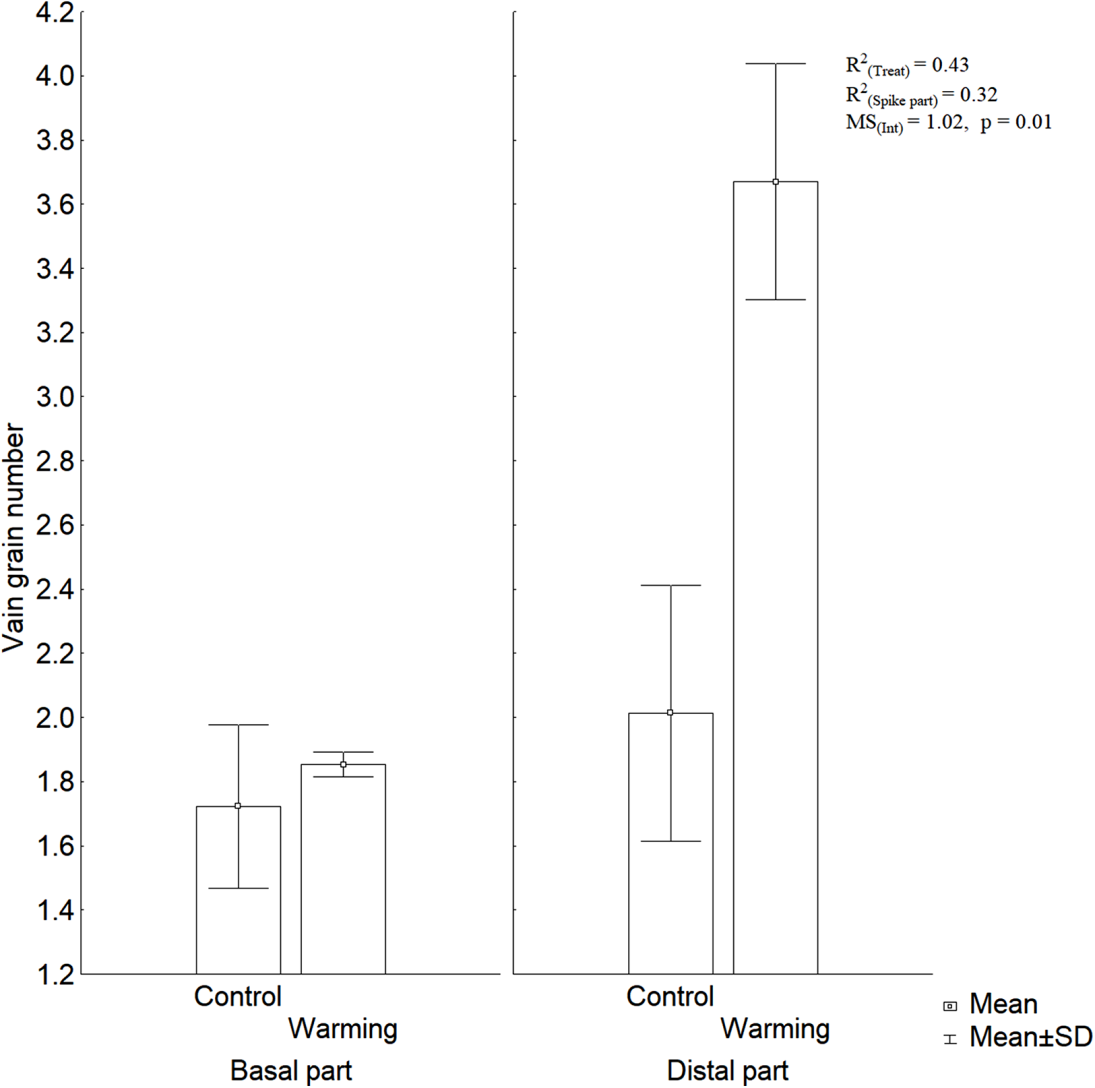
Vain grain number at the basal and distal part of the spikes under warming and control treatments.

Warming explained 43% of the variability observed in the number of vain grains per spike part, in contrast spike part only contributed with 32% to the total variability of vain grains number (Fig. 3). These results show that warming causes an increase in the number of vain grains at the distal spikelets in wheat.

In contrast to other yield components, grain mass (evaluated as 1,000 grains mass) did not vary statistically between the treatments. It neither showed any correlation with yield variation (Table 1. *r* = −0.01; *p* = 0.06).

### Photosynthesis

**Figure 4:**
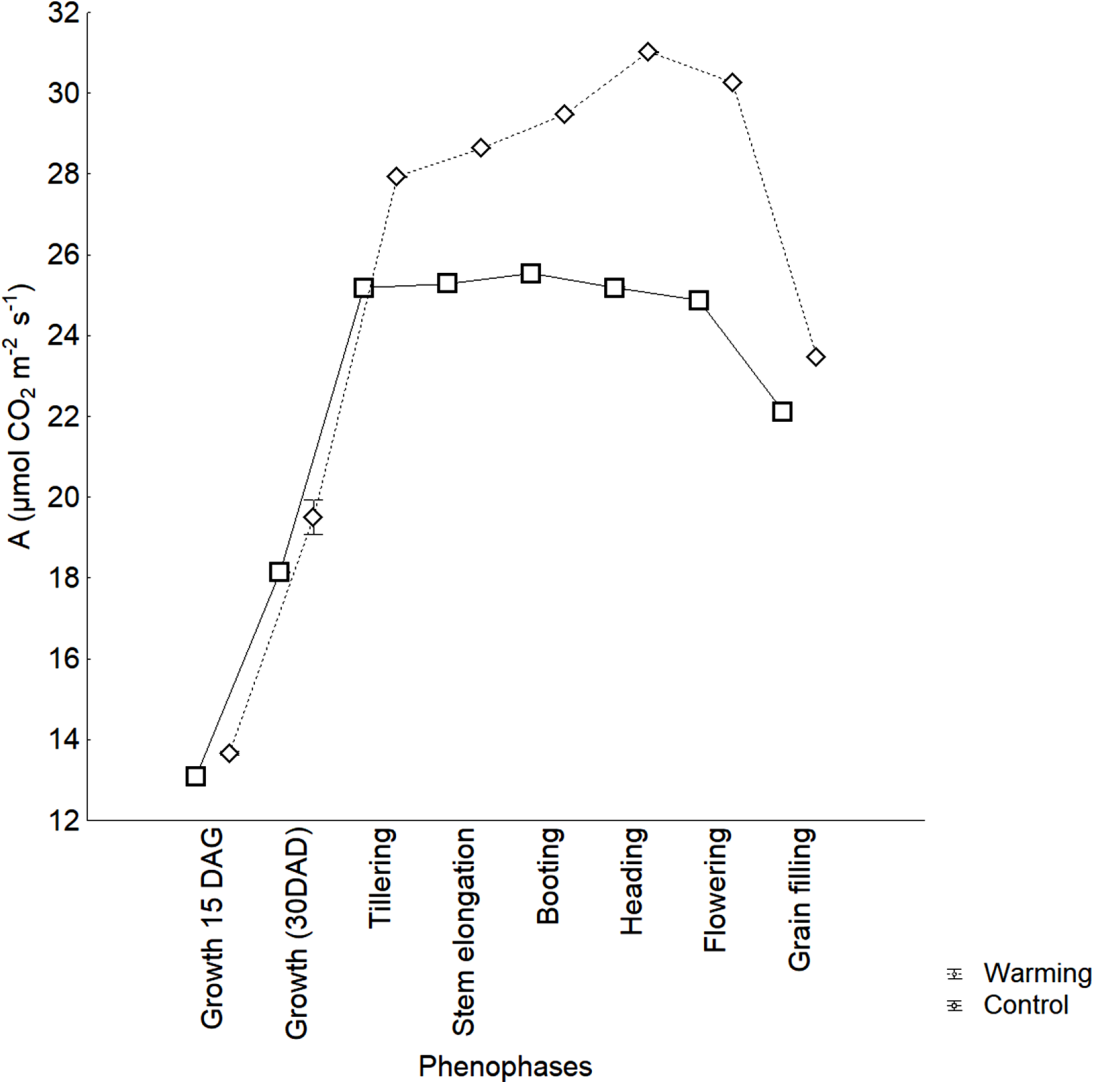
Photosynthetic activity in wheat phenology.

The photosynthetic activity did not show significant differences between the Warming and Control treatments in the initial growing phenophases although it was increasing during the growth phenophases. From Tillering to Heading phenophases, a significant increase of photosynthetic activity was found under the Warming treatment (*p* < 0.0001) (Fig. 4). The maximum photosynthesis value was obtained during Heading phenophase. The Control treatment showed uniform values in all phenophases, from Tillering to Flowering, and a significative reduction during grain filling (24.87 vs 22.11 μmol CO_2_ m^−2^ s^−1^, respectively, *p* = 0.0001). This is a normal process when leaves senescence is starting, and was observed in both treatments. Mean photosynthetic activity was significantly higher in the Warming treatment than in the Control one (25.49 vs 22.42 μmol CO_2_ m^−2^ s^−1^, respectively, *p* = 0.045) and, in contrast to the Control treatment, showed an increasing trend, reaching the maximum value in the Heading phenophase. The observed differences demonstrate that warming has a positive effect on photosynthetic activity over several phenophases.

### Plant height, biomass, and yield

**Figure 5:**
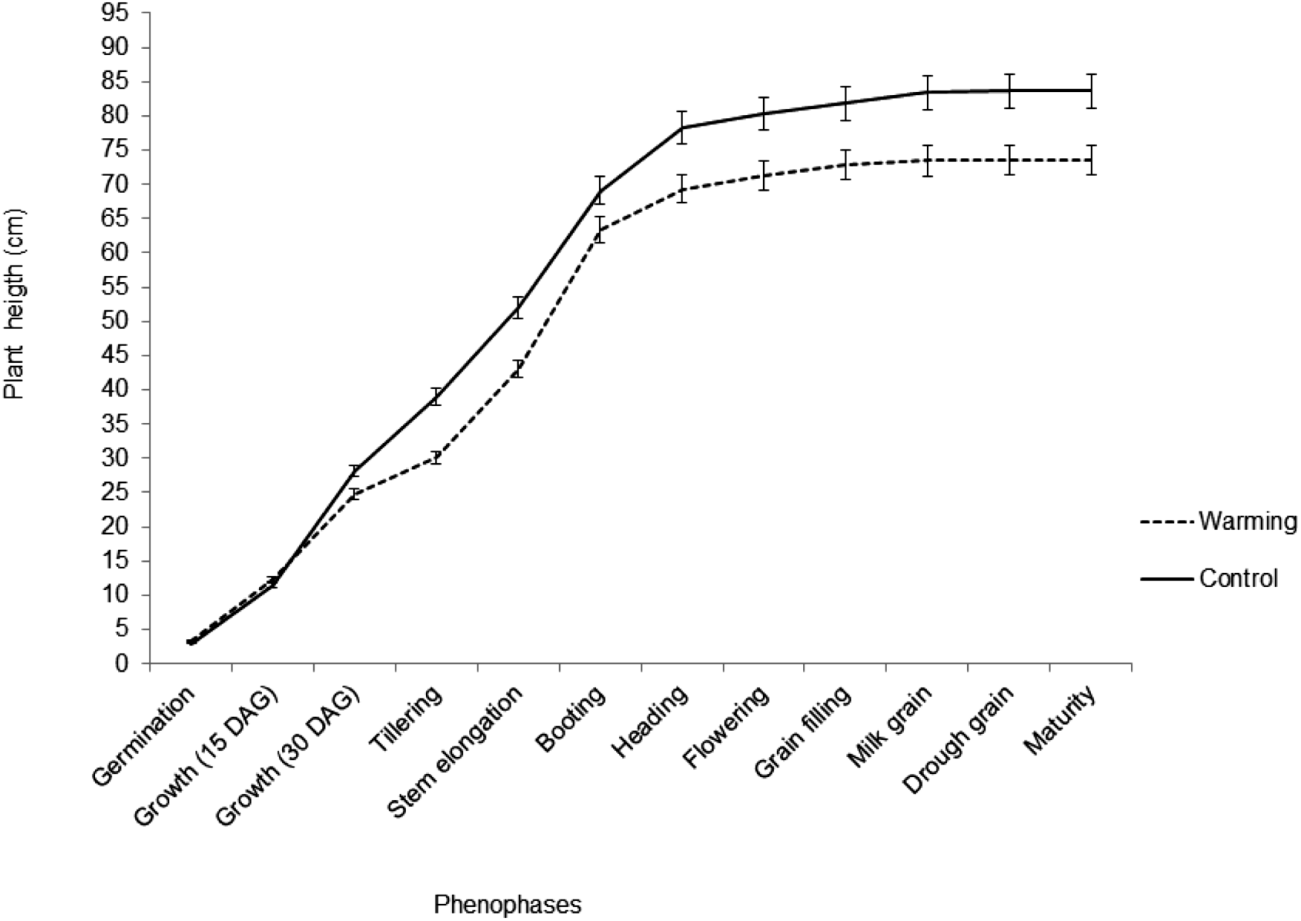
Plant height during the biological cycle of wheat in warming and control treatments; bars represent the standard deviation of the mean.

**Figure 6:**
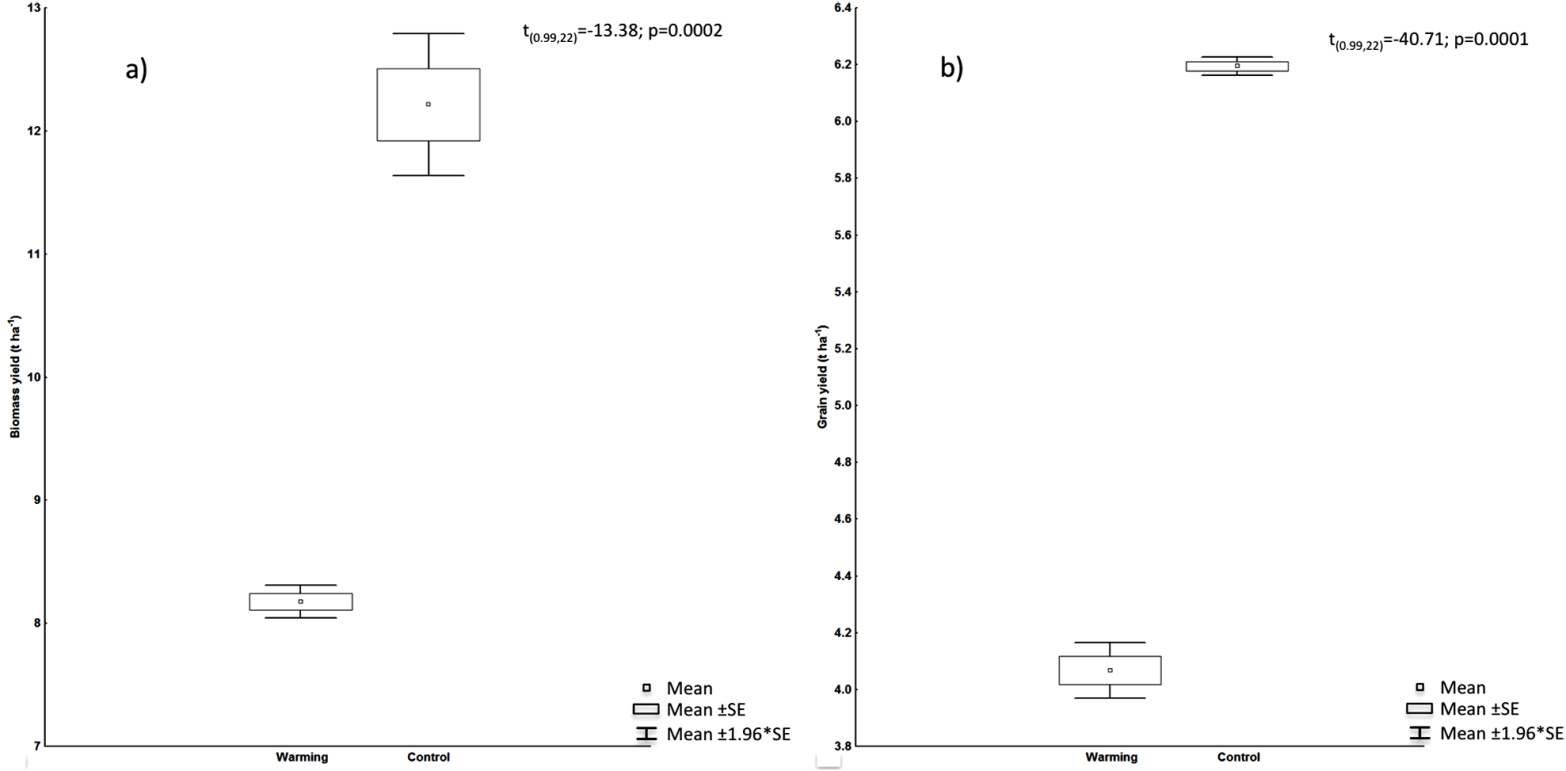
(A) Biomass and (B) grain yield in treatments, *t*-values, and probability.

In the present study, plant height was the most disturbed variable by warming effects, with reductions from the early stages of growth to maturity. During the Heading phenophase, plant height experienced a minor increase due to physiological changes of the apical meristem. Warming caused a reduction of 11.9% on plant height (Fig. 5). Plant height variations correlated negatively with spike length (*r* = −0.89), spike mass (*r* = −0.86), filled grain number (*r* = −0.835), and yield (*r* = −0.94), showing yield the major correlation. Biomass and grain yield showed a strong significant decrease with the warming treatment presenting a reduction of 33% and 34%, respectively (Fig. 6).

### Stress intensity

**Figure 7:**
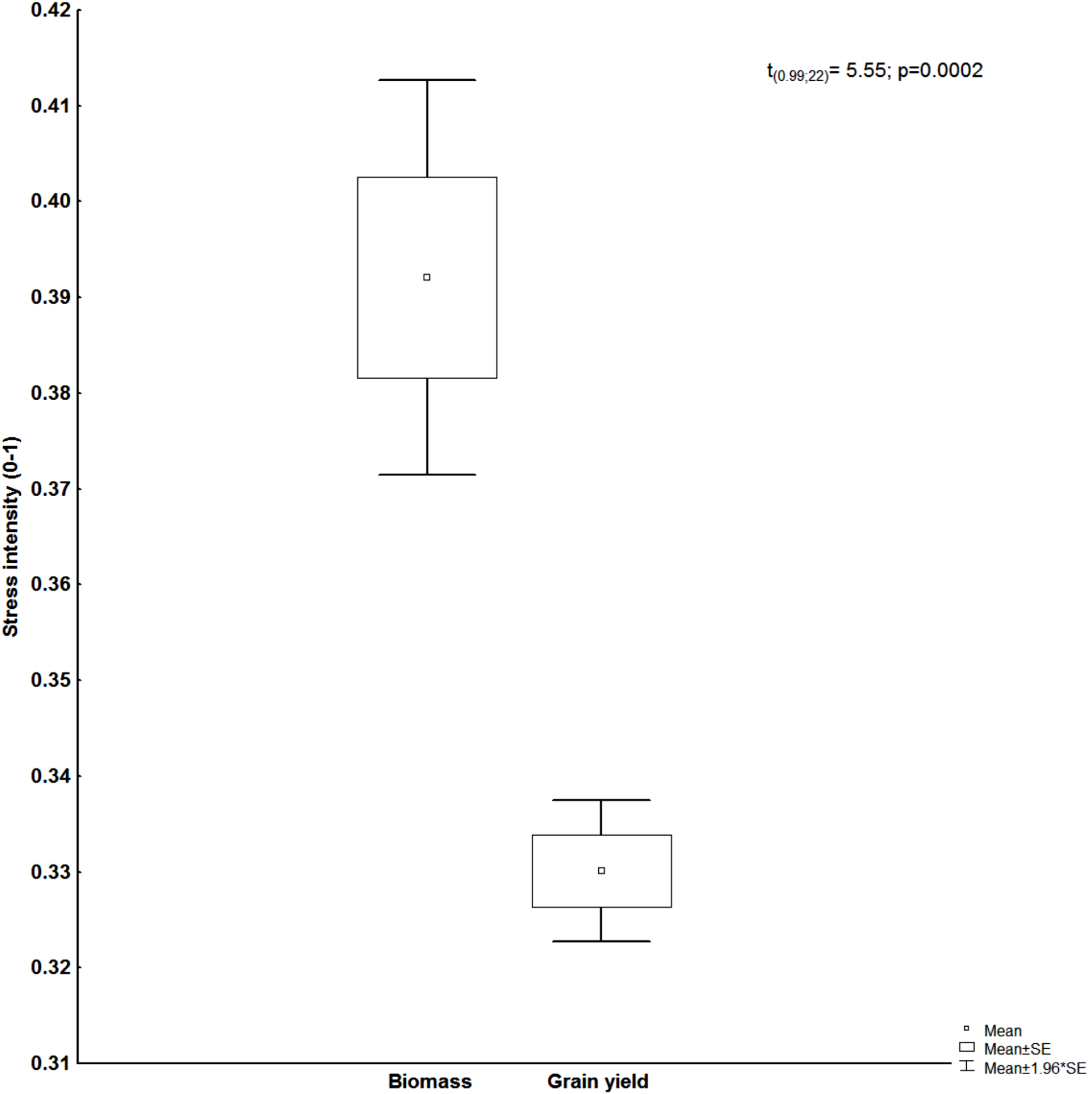
Stress intensity in biomass and grain yield, *t*-values, and probability. Study sites: SE, standard error of the mean.

Warming produced stress intensities of about 39% and 33% in biomass and grain yield, respectively (Fig. 7). These results confirm the negative impact of warming to yield performance on wheat. The obtained values of stress intensity in the present study allowed its classification as severe stress.

## Discussion

The reduction of phenophase occurrence time has been studied in several crops and the effect of this reduction originates from biochemical processes alteration, mainly in the synthesis of substances translocated to the spikes (Shirdelmoghanloo et al., 2016). This reduction in phenophase occurrence time sometimes modifies the expression of structural genes regulators of proteins synthesis, such as glutamine, and its mobilization to the spikes (Royo et al., 2016).

Our results confirm the susceptibility of phenology to warming during reproductive stages. In addition we observed a significant reduction in Stem elongation and Anthesis, emphasizing the negative impact of warming on wheat phenology (Flohr et al., 2017). Some studies have established that in wheat, warming susceptibility appears after the Anthesis (Dias & Lidon, 2009; Prasad, Bheemanahalli & Jagadish, 2017) and it is characterized by an early senescence and poorly filled grains, generating distal spikelets abortion and reducing the individual grain mass, which reduces grain yield (Yang et al., 2017). However, in the present study, this variable (measured in function of mass of 1,000 grains) did not show statistical differences by heat effect (Table 1), although the number of full grains was significantly reduced.

While there is evidence for the change in crop phenology, it is more difficult to find the mechanisms for these changes. Many studies suggest a close relationship between changes in crop phenology and changes in temperature during the growing season. For example, simulation of wheat phenology under expected future climate change suggested that the crop development rate would accelerate due to the effect of higher temperatures, causing a two-week advancement in Anthesis for 2060 (Rezaei, Siebert & Ewert, 2015). Such assumptions, based on mathematical models, provide a possible response of wheat to temperature increase, agreeing with the present study, where a reduction of 10 days at the time of Anthesis and 14 days at harvest, was observed at the warmed plots with respect to the Control.

The spikes length, among different stress types, generally shows a significant variability, result that confirms its polygenic character, which has been reported by some other authors (Jamil et al., 2017; Arya et al., 2017; Mwadzingeni et al., 2017). The number of full grains decrease when the crop experiences a temperature increase, above 1.5 °C, with respect to ambient canopy temperature, particularly immediately before Anthesis (heat wave) (Wheeler et al., 1996; Ferris et al., 1998). It has been also found that the vain grain number of wheat can significantly increase when temperature during mid-anthesis vary in 2 °C during 10 days (Mitchell et al., 1993).

Dwivedi et al. (2017a) pointed out that the higher effects of abiotic stress on grain yield in wheat, are usually due to a reduction in the full grains number and also to an increase of vain grains number (mainly in the distal spikelets) and not to their individual grain mass decrease. Similarly, in the present study, the greater amount of vain grains was found at the distal spikelets (Fig. 3).

The absence of difference on grain mass (evaluated as 1,000 grains mass), between the treatments, agrees with results by Dwivedi et al. (2017a) who found no effect of drought on grain mass. This aspect indicates the capability of CIRNO C2008 variety, used as experimental model at the presently assay, to support seed filling even under an increase of 2 °C with respect to normal environmental canopy temperature. Recently, Argentel Martinez et al. (2018) reported that since 2008 CIRNO C2008 is still the most extended variety in the Yaqui Valley and maintain genetic stability for yield components although climate condition has experience significative change (Lares-Orozco et al., 2016).

Photosynthetic activity contribution to yield is not always proportional (Yang et al., 2017). For example, high temperatures can affect photosynthetic activity indirectly by reducing phenophases duration due to an early senescence. Warming can accelerate senescence in the photosynthetic organs. This is supported in this study by the much stronger reduction in photosynthetic activity from flowering to grain filling, in the Warming treatment in contrast to the pattern showed in the Control (7 vs 2 μmol CO_2_ m^−2^ s^−1^). There are evidences that chlorophyll pigments concentration can be differentially reduced by warming and, although photosynthesis values can be high during certain phenophases, yield may decrease (Yang et al., 2016).

Plant height is an important yield component at the plant and the ecosystem levels. In plant height and grain yield correlation studies, a negative contribution has been obtained when plants height affectations exceeded 10% (Dwivedi et al., 2017a). The negative correlation between plant height and spike length, spike mass, filled grains number, and yield shows that reproductive organ formation is favored over height gain under warming conditions.

The reduction of both biomass and grain yield confirms the negative effects of warming on crop morphology and physiology, despite of high photosynthetic activity in the Warming treatment. In the present study, the high photosynthetic activity contributed to maintain the grain mass, although the number of full grains decreased and the number of vain grains increased (these two variables were the main causes of yield decrease) (Table 1). It is likely that during photosynthesis, under some stressing condition, an important proportion of photoasimilates is used for the synthesis of secondary metabolites, used for plant protection, (Shirdelmoghanloo et al., 2016). Also, during plants exposure to adverse conditions, the macromolecules are degraded to their monomeric bases for osmoregulation and starch remobilization (Chandra et al., 2017).

Warming causes, in some plant species, the production of non-viable pollen (Siebers et al., 2017) turning out difficult fertilization. Sometimes partially filled grains are present which affect grain quality (Nuttall et al., 2017). In the more extreme cases of heat shock during the floral development and the spike filling phenophase, a heterostyly phenomenon is generated, where the development and maturity between stigma and anthers is imbalanced causing fecundation problems (Ferrero, 2014). Although wheat has an encapsulated or covered fecundation, heat can affect the final fertilization process, generating a reduction in grains filled by spike, which is one of the grain yield components of greater contribution to yield losses. Perhaps, in the present assay, warming caused severe damage in pollen quality, generating pollen abortion affecting the final fertilization or infertility (Dwivedi et al., 2017b).

Stress intensity is a response involving physiological and metabolic functions in plants. The duration and intensity of stress during crop growth determines the extent of yield losses (Rezaei, Siebert & Ewert, 2015).

Several studies have suggested a substantial increase in the frequency and magnitude of heat stress, due to climate change, in wheat production of different latitudes. Some of them explicitly investigated possible adaptation strategies against increasing heat stress. However, most of these studies have been conducted by using statistical models, which did not consider changes in crop phenology caused by global warming (Semenov et al., 2014; Rezaei, Siebert & Ewert, 2015). In the present study, canopy temperature of the crop was manipulated by 2 °C to evaluate and correlate physiological and agronomic variables with biomass and grain yield as an integrated warming response of wheat to climate change predicted for 2050 for several latitudes including the Yaqui Valley. These results could serve as a basis for the introduction of tolerant varieties, or in the extreme case, the change on land use, once crops have shown significant yield reductions in relation to their genetic productive potential.

These components response vary according to the stress type, intensity and duration and also depend to a great extent on the tolerance degree of the variety. For this reason, monitoring the response to warming of the available germplasm through phenological, physiological, and agronomic indicators, although is a laborious process (Naik et al., 2016), provide major information over final response of wheat to abiotic stress, and will allow to recommend phenological, yield components characteristic and largely possible progenitors for breading programs for warming climates (Trnka et al., 2017; Yang et al., 2017).

## Conclusion

Warming treatment caused a reduction of phenophases occurrence time starting from stem extension. Such situation generated a significative reduction of the biological cycle of CIRNO C2008 wheat variety of up to 14 days. Nevertheless, the studied variety completed its biological cycle according to Zadoks Decimal Scale.

Plants height, due to warming effect, was reduced significantly and differences were observed on the final phenophases of vegetative stages. Such variable correlated negatively with spikes length, spikes mass, and filled grains number.

Imposed warming reduced biomass and grain yield in 33% and stress intensity generated by the warming treatment were 39.4% and 33.2%, respectively. The differences in stress intensities between biomass and grain yield were based on plant height reduction. Grain mass (or thousand grains mass) did not suffer affectation demonstrating crop capability for remobilization and adequate distribution of elaborated substances in spikes. This result shows that warming would not affect this yield component in future global change scenarios.

## Supplemental Information

10.7717/peerj.5064/supp-1 Supplemental Information 1 Raw data: phenphases, plant height, photosynthesis, yield and grains.

## Additional Information and Declarations

### Data Availability

The following information was supplied regarding data availability: The raw data are provided in a Supplemental File.
